# Single-Cell RNA Sequencing Analysis of Gene Regulatory Network Changes in the Development of Lung Adenocarcinoma

**DOI:** 10.3390/biom13040671

**Published:** 2023-04-12

**Authors:** Dongshuo Yu, Siwen Zhang, Zhenhao Liu, Linfeng Xu, Lanming Chen, Lu Xie

**Affiliations:** 1 Key Laboratory of Quality and Safety Risk Assessment for Aquatic Products on Storage and Preservation (Shanghai), China Ministry of Agriculture, College of Food Science and Technology, Shanghai Ocean University, Shanghai 201306, China; yds290zcf@163.com; 2 Shanghai-MOST Key Laboratory of Health and Disease Genomics (Chinese National Human Genome Center at Shanghai), Institute of Genome and Bioinformatics, Shanghai Institute for Biomedical and Pharmaceutical Technologies, Shanghai 200037, China; zhangsiwen07@163.com (S.Z.); liuzhenhao@sibpt.com (Z.L.); linfneg@163.com (L.X.); 3 College of Food Science and Technology, Shanghai Ocean University, Shanghai 201306, China; 4 Ministry of Education Key Laboratory for Biodiversity Science and Ecological Engineering, Institute of Biodiversity Science, School of Life Sciences, Fudan University, Shanghai 200438, China

**Keywords:** gene regulatory network, lung adenocarcinoma, single-cell transcriptome analysis, macrophage, cell-cell communication

## Abstract

Lung cancer is a highly heterogeneous disease. Cancer cells and other cells within the tumor microenvironment interact to determine disease progression, as well as response to or escape from treatment. Understanding the regulatory relationship between cancer cells and their tumor microenvironment in lung adenocarcinoma is of great significance for exploring the heterogeneity of the tumor microenvironment and its role in the genesis and development of lung adenocarcinoma. This work uses public single-cell transcriptome data (distant normal, nLung; early LUAD, tLung; advanced LUAD, tL/B), to draft a cell map of lung adenocarcinoma from onset to progression, and provide a cell-cell communication view of lung adenocarcinoma in the different disease stages. Based on the analysis of cell populations, it was found that the proportion of macrophages was significantly reduced in the development of lung adenocarcinoma, and patients with lower proportions of macrophages exhibited poor prognosis. We therefore constructed a process to screen an intercellular gene regulatory network that reduces any error generated by single cell communication analysis and increases the credibility of selected cell communication signals. Based on the key regulatory signals in the macrophage-tumor cell regulatory network, we performed a pseudotime analysis of the macrophages and found that signal molecules (TIMP1, VEGFA, SPP1) are highly expressed in immunosuppression-associated macrophages. These molecules were also validated using an independent dataset and were significantly associated with poor prognosis. Our study provides an effective method for screening the key regulatory signals in the tumor microenvironment and the selected signal molecules may serve as a reference to guide the development of diagnostic biomarkers for risk stratification and therapeutic targets for lung adenocarcinoma.

## 1. Introduction

Lung cancer is the most common cancer worldwide [1]. Non-small cell lung cancer (NSCLC) accounts for 85% of lung cancer cases, with a 5-year survival rate of less than 16% [2]. NSCLC mainly contains two subtypes, lung adenocarcinoma (LUAD) and lung squamous cell carcinoma (LUSC). A recent study showed that LUAD and LUSC differed in age, sex, clinical stage, tumor site, histological grade, treatment, and 5-year overall survival, requiring separate analysis of LUAD and LUSC to provide more precise results. The ratio of LUAD to LUSC patients was reported close to 2.44:1, indicating that there are more patients with lung adenocarcinoma. In addition, patients with LUAD are less likely to receive chemotherapy and radiotherapy than those with LUSC, and therefore, screening targets and developing immunotherapy are critical [3]. On the other hand, a growing number of single-cell studies have found a heterogeneous immune landscape between LUAD and LUSC, with the presence of distinct cells and transcriptional modules associated with survival [4,5]. The prognosis for LUAD patients and treatment decisions are based on the clinical stage of the disease at the time of diagnosis. Tumor progression is a continuous process of change; it is more important to accurately assess lung adenocarcinoma from normal, early to advanced stages. At present, the main treatment for early LUAD (tLung-tumor lung) is surgical resection with lobectomy [6]. The treatments for advanced LUAD (tL/B-tumor lung/with brain metastasis) include targeted therapies and chemo-radiotherapy [7]. Current clinical techniques may not provide complete information about the molecular characteristics to distinguish between early and advanced LUAD [8], hence, single-cell RNA sequencing was used to assess the intratumor heterogeneity of LUAD and proved essential for understanding the biological nature and the developmental status of the disease [9].

Technological advances have allowed single-cell analysis to reveal that cell-cell communication plays a crucial role in numerous biological processes that utilize a dynamic network to support communication and cooperation between cells, e.g., tissue homeostasis [10,11], cell development [12,13], disease pathogenesis and progression [14,15], and therapy resistance [16]. Cell-cell communication in tumor microenvironments (TMEs) drives cancer progression and influences the response to existing therapies [17]. Macrophages have long been considered to be important immune effector cells in TMEs and play an important role in regulating innate and acquired immunity, healthy tissue homeostasis and vasculogenesis [18]. However, macrophages are highly plastic in the TME. For example, tumor cells can take advantage of macrophage plasticity to reshape them into an immunosuppressive phenotype (e.g., tumor-associated macrophage, M2-like immunosuppressive phenotype, etc.) [19]. M2 macrophage-derived exosomes could be taken up by tumor cells to promote cell migration, invasion, and angiogenesis [20]. Tumor-associated macrophages (TAMs) have increasingly been recognized as predicting a lung adenocarcinoma (LUAD) prognosis [21]. The tumor microenvironment of LUAD is complex, including the immune activation microenvironment and the immune suppression microenvironment [22]. In these two different tumor microenvironments, macrophages with different functions play a central role in the heterogeneity of the LUAD immune microenvironment [5]. For example, AT2-like malignant cells with high expression of ANXA1, MDK and FN1 in LUAD may be responsible for the recruitment of macrophages with high expression of FPR1 and SORL through ligands [23]. In addition, the characteristics and components of antigen-presenting macrophages are continuously reduced in LUAD, and the communication between tumor epithelium and macrophages expressing CX3CR1 cognate receptors is increased [24]. Therefore, understanding the relationship between tumor cells and macrophages in the microenvironment is crucial for the occurrence and development of LUAD. At present, intercellular communication is mostly based on biomolecular interactions, e.g., ligand-receptor link, ligand-target link. Therefore, there may be errors in the predicted outcomes of these different cell communication prediction tools. A recent study has revealed great diversity between the different lesions of LUAD at the single-cell level through cell-cell communication, but cell interaction molecules cannot be experimentally verified [25]. Thus, improving the credibility of cell-cell communication is important for the understanding of the microenvironmental alterations and immune responses in lung adenocarcinoma.

In this study, we used single-cell RNA sequencing data of three LUAD states (nLung-normal Lung, tLung, tL/B) to explore the association between cell-cell crosstalk and lung cancer progression based on differences in the tumor-macrophage communication gene regulatory network signals. Further, we found that these signals, exhibiting significant differences in gene regulatory networks, played a key role in the transformation of macrophages into immunosuppressive phenotypes using pseudotime analysis, and may affect the occurrence and development of LUAD. In addition, we found that the tumor microenvironment and gene regulatory network of lung squamous cell carcinoma retained similar features but were different from LUAD. Overall, we demonstrate that communication between tumor cells and macrophages alters the functional status of the macrophages; the resulting regulatory signals were significantly associated with poor prognosis in lung adenocarcinoma patients.

## 2. Materials and Methods

### 2.1. Data Collection and Preprocessing

To describe the composition and functional status of lung adenocarcinoma (LUAD) during tumor progression, single cell transcriptome profiles from the lung tissues of distant normal (nLung), early LUAD (tLung) and advanced LUAD (tL/B) were collected from the dataset GSE131907 in the GEO database [26]. Sequencing data were mapped to the GRCh38 human reference genome using the Cell Ranger toolkit (version 2.1.0). Quality measures were applied to the raw gene-cell-barcode matrix for each cell based on: mitochondrial genes (≤20%, unique molecular identifiers (UMIs), and gene count (ranging from 100 to 150,000 and 200 to 10,000). In total, 42,995 cells of lung tissues from distant normal (nLung), 45,149 cells of lung tissues from early LUAD (tLung) and 12,073 cells of lung tissues from advanced LUAD (tL/B) were merged.

To validate the regulatory network changes during lung adenocarcinoma development another set of single cell transcriptome data (GSE123902 [27], GSE117570 [28], GSE148071 [29]) was used as a validation dataset. After quality control, a total of 34,920 cells were selected for subsequent analysis (including 15,701, 14,984, and 4235 cells from distant normal regions of lung samples, early LUAD, and advanced LUAD). The single-cell data of the validation group were used to construct the gene regulatory network through the same analysis process. In addition, in order to observe the heterogeneity of LUAD and LUSC, a LUSC’s single cell transcriptome data (http://lungcancer.chenlulab.com (accessed on 22 March 2023)) was analyzed through the same pipeline. A total of 18,117 cells were selected for subsequent analysis (including 6338, 7918 and 3861 cells from normal tissue samples of LUSC, early LUSC and advanced LUSC) after quality control.

### 2.2. Data Integration, Unsupervised Dimensional Reduction and Clustering, and Cell Type Identification

scRNA-seq data were normalized using the “NormalizeData” function; and were scaled using the “ScaleData” function. The top 3000 highly variable genes were identified using the “FindVariableFeatures” function. Next, we used the “RunPCA” function to reduce the dimension of the scRNA-seq data. To integrate cells within a shared space from different datasets for unsupervised clustering, “RunHarmony” of Harmony (version 0.1.0) [30] was used to identify anchors and run integration steps and eliminate batch effects. Pcs were selected by ranking the principal components using the ElbowPlot function in the Seurat R package. This function works by randomly permuting a subset of data, and calculating projected PCA scores. When an inflection point reached the 20th pc, then, the first 20 principal components (PCs) were utilized in UMAP (uniform manifold approximation and projection) analysis using “RUNUMAP”. Subsequently, a single cell map at 0.2 resolution was presented by the “FindClusters” function. Further, the “FindAllMarkers” function was used to detect gene expression markers. The above analysis was performed using the Seurat (version 4.1.1) [31] R package. Afterwards, we used the R package SingleR (version 1.6.1) [32], CellMarker dataset [33] and marker genes of cells to annotate cell types in our study.

### 2.3. Identification of Malignant Cells from Patients with Adenocarcinoma of the Lung

In order to isolate malignant tumor cells from patients with lung adenocarcinoma, CNV aberrations were inferred from the patterns of chromosomal gene expression by inferCNV (version 1.8.1) [34]. Since the data were 10× Genomics single-cell data, usually the cutoff is set = 0.1, denoise = T. The expression profiles of lung tissues from distant normal regions were used as a reference, the early LUAD and the advanced LUAD were used as the observation group.

### 2.4. Functional Enrichment Analysis

R package clusterProfiler (version 4.0.5) [35] was used for Gene Ontology (GO) annotation and enrichment analyses. *p*-value (p.adjust value) was calculated using the Benjamini and Hochberg method [36]. A p.adjust value < 0.05 was considered significant. In the enrichment analysis related to the regulatory network, *p*-values were not adjusted in order to retain more variables for multivariate analysis. Graphic visualization was enabled using ggplot2 (version 3.2.1).

### 2.5. Survival Analysis of the Proportion of Macrophages in Patients with Lung Adenocarcinoma

The LUAD mRNA expression data and associated clinical data in TCGA were downloaded from the UCSC Xena (http://xena.ucsc.edu/ (accessed on 10 July 2022)) database. The proportional distribution of 22 infiltrated immune cell types in patients was obtained by CIBERSORT [37] in TCGA-LUAD data. The patients were assigned to one of two groups (high risk and low risk) according to the proportion of infiltrated immune cells. Then, the relationship between the proportion of macrophage cells with survival was evaluated using the R package survival (version 3.3.1) [38]. The “surv_cutpoint” algorithm of R package survminer (version 0.4.9) was used to calculate the optimal threshold, and all survival analyses adopted this approach. In order to retain more variables for multivariate analysis, *p*-values were not adjusted.

In order to confirm the effect of tumor ligand signaling in the regulatory network on the occurrence and development of lung adenocarcinoma, the GEPIA2 [39] tool was used to evaluate the relationship between ligands in the gene regulatory network and the survival of patients with lung adenocarcinoma.

### 2.6. The Gene Regulatory Network of Tumor Cell-Macrophage Interaction Construction

First, the R package, Nichenet (version 1.0.0) [40], was used to infer the gene regulatory network of tumor cell-macrophage interactions. Malignant cells were designated as sender cells, macrophages were designated as receiver cells, “top_n_ligands” = 30, “condition_reference” was designated as nLung, “condition_oi” was designated as tLung, and then, through the function of “nichenet_seuratobj_aggregate”, ligand-target pairs were identified. Further, ligand-receptor (L-R), receptor-transcription factor (R-TF), transcription factor-target (TF-target) were found by the “get_ligand_signaling_path” function. Using the above steps, gene regulatory networks linking nlung and tLung and linking tLung and tL/B were identified second. CellPhoneDB (www.cellphonedb.org (accessed on 7 August 2022)) [41] was used to identify ligand-receptor pairs between tumor cells and macrophages, and those pairs with mean values greater than 0 were reserved. The ligand-receptor pairs in the second step were verified using Nichenet, and these verified interaction pairs and their downstream signaling molecules were retained. Third, pySCENIC (version 0.11.2) [42] was used to identify the TF-target of the macrophages, retaining those regulatory chains in the TF-targets that overlap with the previous step’s regulatory network. Finally, the differentially expressed genes (DEGs) were found using the “Findmarker” function of Seurat to screen the gene regulatory network signals (Section 3.3).

### 2.7. Immune Cell Trajectory Is Constructed by Regulating the Signaling Molecules of the Network

In order to validate the signals (receptor, TFs, target) of gene regulatory network 4.0 influenced macrophages during LUAD progression, monocle (version 2.14.0) [43] was used to analyze the gene expression matrix with macrophage cells. We used the signals (receptor, TFs, target) of the gene regulatory network 4.0, to sort cells in pseudotime order. “DDRTree” was applied to reduce the number of dimensions and the visualization functions “plot_cell_trajectory” was used to plot the minimum spanning tree on cells. Finally, cells of nLung were defined as the starting point through “orderCells” function.

## 3. Results

### 3.1. Single-Cell Expression Atlas and Cell Typing in Normal Lung, Early and Advanced LUAD Tissue Samples

**Figure 1:**
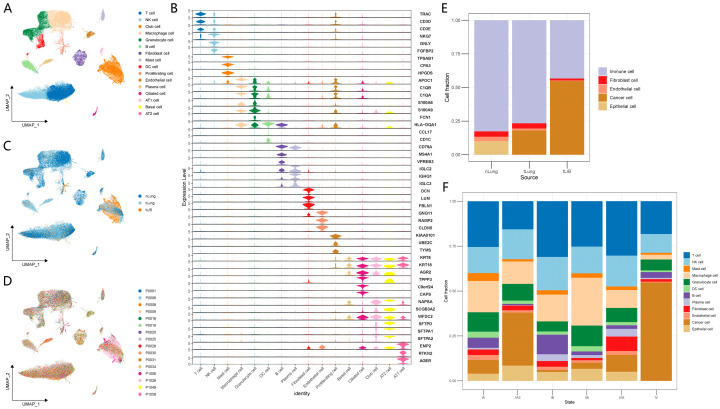
A single-cell atlas of nLung, tLung and tL/B. (**A**) The UMAP plot of high-quality cells to visualize cell-type clusters. (**B**) Stacked violin plots displaying markers of the expression across sixteen cell types. (**C**,**D**) The UMAP plot cell distribution in tissue source and patient source. (**E**,**F**) Proportion of cells in nLung, tLung and tL/B and different stages (IA, IA3, IB, IIA, IIIA, and IV).

To describe the composition and function of cells of LUAD during their different statuses, single-cell transcriptome datasets were collected from LUAD with 26 tissue samples from 16 patients, including 11 samples of distant normal lung tissues, 11 early LUAD samples, and 4 advanced LUAD samples (Table S1). After quality control, a total of 100,210 cells were selected for subsequent analysis (including 42,995, 45,149, and 12,073 cells from distant normal tissue of lung samples, nLung; early LUAD, tLung; and advanced LUAD, tL/B, respectively). The R package “Harmony” was used to integrate data from different samples. After reduction and clustering, cells were divided into 16 clusters. The results of the cell types from the R package SingleR were kept as a reference (Figure S1A), and clusters were annotated (Figure 1A), according to the expression level of marker genes (Figure 1B). A total of 16 cell types were annotated: 8 types of immune cells (including T cells, NK cells, macrophage cells, granulocyte cells, DC cells, mast cells, B cells, and plasma cells), 2 types of stroma cells (including fibroblast cells and endothelial cells), and 6 types of epithelial cells (including AT1 cells, AT2 cells, club cells, ciliated cells, basal cells, and proliferating cells). After integration, the cells grouped primarily by dataset were combined (Figure 1C,D).

The epithelial cells of LUAD exhibit significant heterogeneity [44]. The R package infercnv was used to identify non-malignant cells and malignant cells. The hallmarks of cancer cells are aneuploidy and chromosomal copy number variations (CNV) [45]. The expression profiles of distant normal regions of lung tissues were used as a reference, and the early LUAD and the advanced LUAD were used as the observation group; we found that the copy number of cells provided by lung tissues of early LUAD and advanced LUAD in club cells, ciliated cells, AT1 cells, and proliferating cells exhibited significant changes (Figure S1B). The lack of basal cells and AT1 cells in nLung, and the presence of tumor biomarkers of LUAD [46] were observed in potentially malignant cells. Basal cells and AT2 cells were both highly expressed tumor markers of LUAD (Figure S1C). In addition, basal cells are highly correlated with proliferating cells, and AT2 cells are highly correlated with club cells (Figure S1D). Therefore, we speculate that club cells, ciliated cells, AT1 cells, proliferating cells, basal cells, and AT2 cells are malignant cells.

Overall, the distribution of cells in different patients, different sources, and different pathologies was observed. While tumor cell numbers increase as the disease progresses, the largest proportion of tumor cells exists in tL/B, while immune cells in tL/B are depleted (Figure S1E). The increase in tumor cells may be related to the decrease in immune cells. The proportion of fibroblast cells and endothelial cells did not appear to change. There was no significant change in cell number for different pathological stages (Figure 1F).

### 3.2. The Role of Macrophages in the Immune Microenvironment

**Figure 2:**
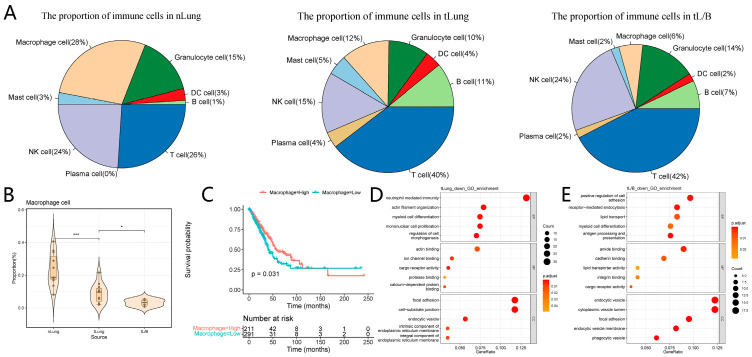
Biological function of a macrophage cell and its impact on patient survival. (**A**) Immune cell composition in nLung, tLung, and tL/B. (**B**) Box plot of the cell percentage of macrophage cells in the three sources: distant normal tissue (nLung), early LUAD (tLung), and advanced LUAD (tL/B). * *p* adjusted < 0.05, and *** *p* adjusted < 0.001 (t.test). (**C**) Correlation between the proportion of macrophages with survival rate; orange represents high ratios and green represents low ratios, (*p* = 0.031). (**D,E**) The GO function enrichment analysis results of downregulated genes of the early LUAD (tLung) (distant normal tissue (nLung) as the control), and downregulated genes of the advanced LUAD (tL/B) (early LUAD (tLung) as the control), *p* adjust < 0.05.

From the above, we have found that the number of immune cells was reduced and the number of tumor cells increased as cell status progresses from normal to early LUAD and advanced LUAD (tLung and tL/B). We then further analyzed the composition of immune cells in the three disease stages and found that macrophages accounted for about 28% of the largest proportion of immune cells in nLung. T cells accounted for the largest proportion of immune cells in tLung and tL/B. Plasma cells were only found in tLung and tL/B (Figure 2A). Further, we studied the proportion of each immune-cell type in the three stages of LUAD, distant normal tissues of lung (nLung), early LUAD (tLung), and advanced LUAD (tL/B). Compared with the cells in nLung, the proportion of B cells and plasma cells was observed to increase in tLung (*p* < 0.05); the proportion of NK cells, macrophages and granulocytes was observed to decrease in tLung (*p* < 0.05). Compared with the cells in tLung, macrophage cells, plasma cells, and DC cells were observed to decrease in tL/B (*p* < 0.05) (Supplementary Figure S2A,B). Interestingly, the changes in macrophages were more pronounced than in other immune cell types (Figure 2B). Macrophages are first responders for the immune system, initiating and coordinating a multipronged immune response [47]. Therefore, we speculate that the decrease in macrophages may be an important reason for the occurrence and development of LUAD. Thereafter, we observed the relationship between the number of macrophages and the survival rate of patients, a Cox proportional hazards model was applied, and the patients with a lower proportion of macrophages showed a lower chance of survival (Figure 2C and Figure S3A). Survival analysis confirmed our speculation.

The above study found that the decrease in macrophages may be associated with the occurrence of lung adenocarcinoma. Moreover, changes in the function of macrophages may also affect the development of lung adenocarcinoma during the process that decreases the number of macrophages. Thus, the “FindMarker” function of the package Seurat was used to identify the differentially expressed genes in the macrophage cell population between nLung and tLung and tL/B. In tLung, the upregulated genes were associated with the positive regulation of the immune system process and its response to hypoxia (e.g., T-cell activation, lymphocyte proliferation and response to decreased oxygen levels), whereas the genes downregulated primarily belonged to proliferation-related pathways (e.g., mononuclear cell proliferation, myeloid cell differentiation). According to the results of functional analysis, macrophages from tLung patients are in a state of promoting immune activation compared with nLung people, and the downregulation of proliferation-related function may be related to the decrease in the proportion of macrophages. The upregulated genes in tL/B were related to the response to positive regulation of angiogenesis and vasculature development, and the downregulated genes in tL/B were related to the positive regulation of cell adhesion, antigen processing, and T-cell activation through the GO function enrichment analysis (Figure 2E and Figure S3B). The results showed that macrophages are in an immunosuppressive state in the tL/B stage and may promote tumor growth by promoting the increase in angiogenesis and vasculature development. In conclusion, during the development of LUAD, the proportion of macrophages gradually decreases, and their function evolves from immune activation (trying to fight against tumors) to immunosuppression, which may create an environment suitable for tumor growth. These results suggest that macrophages may be regulated by tumor cells during the transformation from tLung stage to tL/B stage.

### 3.3. Construction of the Regulatory Network of Macrophages Regulated by Tumor Cells

**Figure 3:**
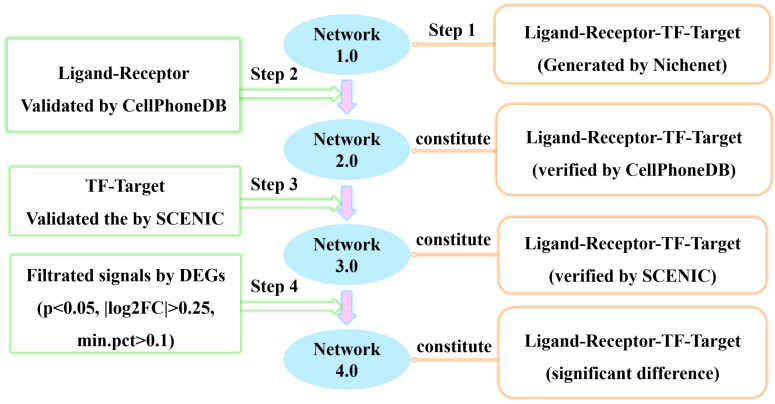
The gene regulation network construction process.

Macrophages exhibit strong plasticity and functional heterogeneity, and their phenotypic transformation is complex [48]. A recent study found that tumor cells can alter the macrophage phenotype. To further investigate which molecules of tumor cells might mediate the change in macrophage cells, we constructed the gene regulatory network comprised of four elements: ligand-receptor-transcription factor (TF)-target. Gene regulatory networks were constructed in four steps (Figure 3). First, we applied Nichenet to obtain intercellular signal transduction networks and constructed ligand-receptor-TF-target links (Network 1.0); second, ligand-receptor (L-R) pairs of the gene regulatory network were verified by CellPhoneDB, the overlapping ligand-receptor pairs and their downstream signals were retained (Network 2.0); third, SCENIC was used to verify TF-target pairs of Network 2.0. The verified TF-targets of Network 2.0 were preserved (Network 3.0); fourth, differentially expressed genes (DEGs) were used to filter important signals of Network 3.0 (Network 4.0). After multiple screening of regulatory networks, the number of signaling molecules is reduced, which means that the resulting overlapped common filter, derived from multiple communication tool analyses, can reduce the false positive results with the use of a single tool.

**Figure 4:**
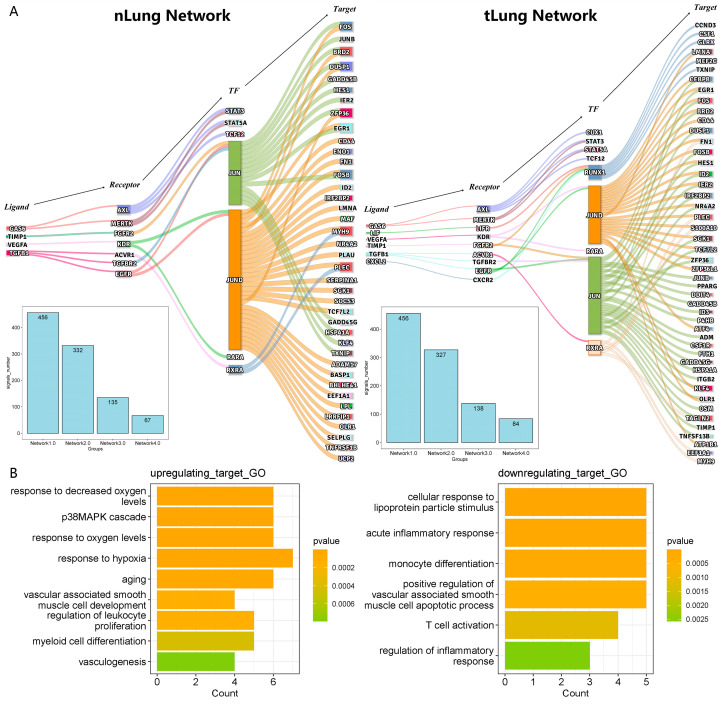
Changes in regulatory networks in nLung and tLung. (**A**) Gene regulatory network of distant normal tissue (nLung) and gene regulatory network of early-stage LUAD (tLung). The network consists of ligand-receptor-TF (transcription factor)-target. (**B**) The GO function enrichment analysis results of targets of tLung network.

The construction of the gene regulatory network may further improve the accuracy and integrity of the signal transduction process. By comparing the networks of nLung and tLung, it was found that multiple signaling pathways were involved. In Network 4.0, 67 signals of nLung and 84 signals of tLung were retained. Regulatory links based on four ligands (GAS6, TIMP1, VEGFA, TGFB1) were found in nLung Network 4.0 (Figure 4A). However, in tLung Network 4.0, a total of six ligands (GAS6, TIMP1, VEGFA, TGFB1, LIF, CXCL2) were included. The functional analysis illustrated that the upregulated signals (receptor, TF, target) in tLung were related to the immunological effect and some upregulated targets of tLung were related to the response to decreased oxygen levels (Figure 4B and Figure S4B). For example, after TIMP1 interacts with FGFR2 (receptor), the expression of ADM mediated by the transcription factor JUN is elevated. A typical hypoxic factor of ADM has been used as an important factor in the tumor microenvironment for predicting clinical prognosis in lung adenocarcinoma [49]. The downregulated molecules in tLung were related to inflammatory response. VEGFA regulates the reduced expression of CD44 through signal transduction. Alveolar macrophages (AMs) are CD44-expressing cells located in the alveolar space that maintain lung homeostasis. When CD44 is downregulated, AMs are unable to bind glycosaminoglycans and hyaluronan, which reduces their viability and leads to a decrease in the number of AMs in the lung [50]. It is worth noting that a regulatory network dominated by LIF-LIFR was found in the tLung network. Recent studies have implicated LIF-LIFR signaling in playing a key role in tumor growth, progression, metastasis, stemness and therapy resistance; the LIF-LIFR axis may be considered as a promising clinical target for cancer therapy [51]. 

**Figure 5:**
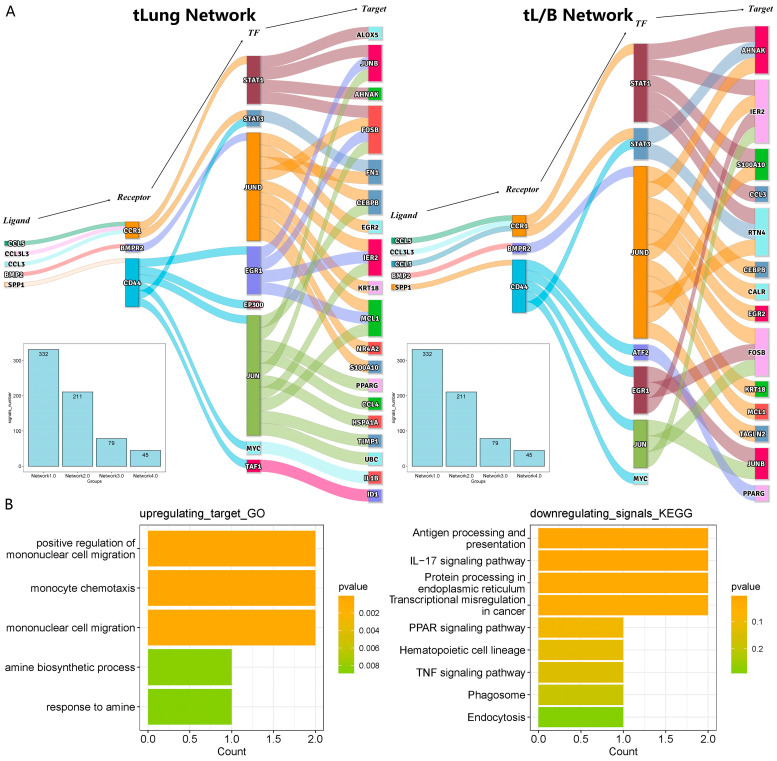
Changes in regulatory networks in tLung and tL/B. (**A**) Gene regulatory network of early-stage LUAD (tLung) and gene regulatory network of advanced-stage LUAD (tL/B). The network consists of ligand-receptor-TF (transcription factor)-target. (**B**) Left: the GO function enrichment analysis results of targets of tLung network. Right: the KEGG function enrichment analysis results of signals (receptor, TF, target) of tLung network.

In the comparison of the network of tLung and tL/B, 45 signals of tLung and 38 signals of tL/B were retained. Regulatory links based on five ligands (CCL5, CCL3L3, CCL3, BMP2, SPP1) were found in Network 4.0 of tLung and tL/B (Figure 5A). The functional analysis illustrated that the downregulated signals (receptor, TF, target) in tL/B were related to immune response (e.g., antigen processing and presentation, IL-17 signaling pathway), and the upregulated targets of tL/B were related to mononuclear cell migration (Figure 5B and Figure S4D). BMP2 regulates the increased expression of EGR2 through signal transduction; EGR2 was highly expressed in tL/B and was reported to be a conserved marker of alternately activated macrophages (M2 macrophages) [52].

In conclusion, the construction of the gene regulatory network improves the accuracy and integrity of deciphering signaling molecules in intercellular communication. In addition, with respect to the progression of lung adenocarcinoma, the changes in the regulatory network may be closely related to the changes in macrophage cells. These ligand signals of tumor cells may be crucial for the occurrence and development of lung adenocarcinoma.

### 3.4. The Signal Changes in the Gene Regulatory Network Are Related to the Change in Macrophage State 

**Figure 6:**
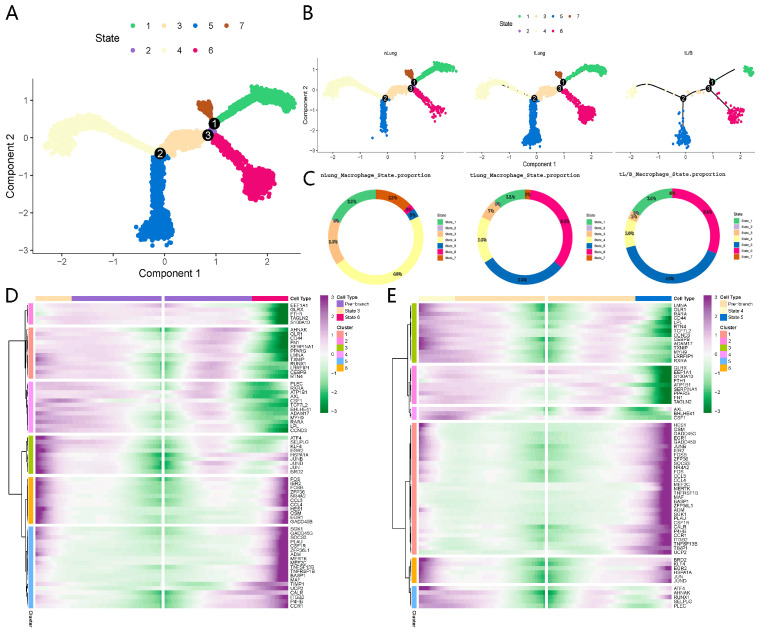
Macrophage trajectory. (**A**) Pseudotrajectory of macrophages. (**B**) Pseudotrajectory of macrophages in source. (**C**) Proportion of macrophages in different states of the three sources (nLung, tLung, tL/B). (**D**) Heatmap shows the gene expression dynamics of branch 3 in the macrophage group. Genes (rows) of the gene regulatory network are clustered and cells (columns) are ordered according to the pseudotime development. (**E**) Heatmap shows the gene expression dynamics of branch 2 in the macrophage group. Genes (rows) of the gene regulatory network are clustered and cells (columns) are ordered according to the pseudotime development.

To further verify the relationship between the changes in signaling molecules during disease progression and macrophage status, we used signal molecules in the gene regulatory network as features for the trajectory analysis of macrophage cells. The R package “monocle” was used to sort individual cells by these signals to construct the tree-like structure of the entire lineage differentiation trajectory (Figure 6A). Macrophages were divided into seven states (state 1, 2, 3, 4, 5, 6 and 7). The macrophages in nLung were dominated by state 3 and state 4; the macrophages in tLung and tL/B were dominated by state 5 and state 6 (Figure 6B,C). During the development of lung adenocarcinoma, the proportion of macrophages in states 3 and 4 mainly decreased, while the proportion of macrophages in states 5 and 6 increased, on the premise that the overall proportion of macrophages decreased; this means that the immune effector function of macrophages may be changed (Figure 6C). Further, we mapped macrophage states 3, 4, 5 and 6 in Seurat and found that states 3 and 4 were concentrated with alveolar macrophages, while states 5 and 6 were concentrated with tumor-associated macrophages (Figure S6D,E). Pathway analysis indicated that the signaling pathways involved in the antigen processing and presentation and MHC class II protein complex binding were enriched in state 3 and state 4 (Figure S5A,B). Macrophages, as typical antigen-presenting cells, were enriched in the antigen processing and presentation of exogenous peptide antigen via MHC class II [53]. Highly expressed genes in state 6 were related to the response to oxidative stress. Pathway analysis suggested that cells in state 5 were dependent on negative regulation of the immune system process (Figure S5C). Furthermore, the aberrantly expressed signaling molecules in the gene regulatory network were significantly expressed in macrophage state 5 and state 6 (Figure 6D,E). For instance, CD44 expression was downregulated and ADM expression was upregulated in state 5 and state 6. 

The results of the pseudotime analysis of the macrophages further confirmed that signal molecules in the regulatory network are key factors affecting the change in the functional state of macrophages, and the abnormal expression of ligand signals (TIMP1, VEGFA, TGFB1, LIF, CCL3L3, BMP2, SPP1) in tumor cells may be an important reason for the transformation of macrophages into an immunosuppressive state.

### 3.5. The Independent Validation Set of Lung Adenocarcinoma (nLung, tLung and tL/B) Was Used to Verify the Regulatory Network

**Figure 7:**
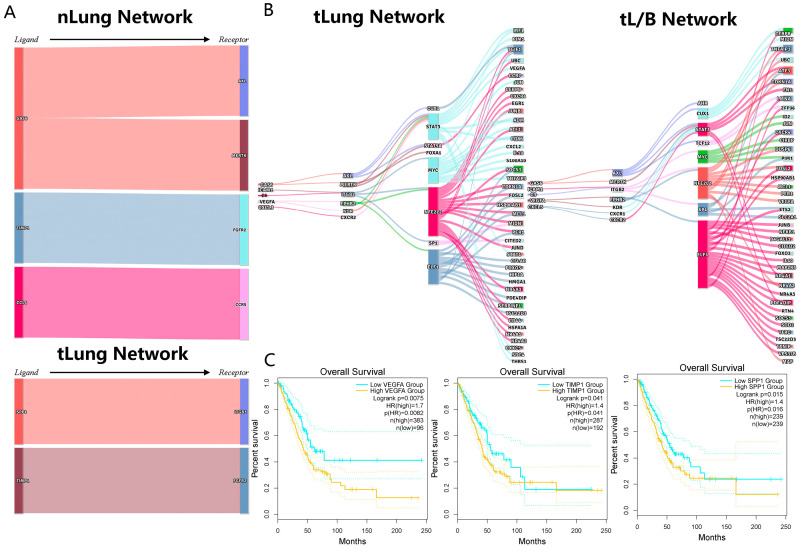
Validation of regulatory networks. (**A**) Gene regulatory network of distant normal tissue (nLung) and gene regulatory network of early-stage LUAD (tLung). (**B**) Gene regulatory network of early-stage LUAD (tLung) and gene regulatory network of advanced-stage LUAD (tL/B). (**C**) Kaplan-Meier estimates of overall survival from LUAD patients in TCGA based on ligands (VEGFA, TIMP1, SPP1).

To further verify the reliability of the gene regulatory network screening method and the consistency of signal molecules, we collected an independent lung adenocarcinoma single-cell transcriptome dataset as a validation set to construct its gene regulatory network. In the independent validation set, we found regulatory chains with GAS6, TIMP1, SPP1, and VEGFA as ligands, which was consistent with our findings above, indicating that these regulatory relations may be ubiquitous in tumor cells and macrophages (Figure 7A,B). Due to the small number of cells in the validation set, some signals were not validated. Next, a survival analysis of the validated ligands revealed that the high expression ligand signals VEGFA, TIMP1, and SPP1 were significantly associated with a lower chance of survival in patients with lung adenocarcinoma (Figure 7C). The upregulation of VEGFA can induce angiogenesis and recruit monocytes to the tumor niche, which are then transformed into tumor-associated macrophages (TAM) in the tumor to participate in tumorigenesis [54]. The upregulation of TIMP1 can regulate cell behavior by inducing signaling pathways involved in cell growth, proliferation and survival [55], and previous studies have found that TIMP1 may be a potential biomarker for the pathogenesis of LUAD [23]. The upregulation of SPP1 can promote the proliferation, migration and invasion of lung cancer cells, and increase the resistance of lung cancer to cisplatin [56]. Overall, VEGFA-, TIMP1, and SPP1-mediated regulatory networks may not only be the main cause of macrophage changes, but also these three signals may be markers of malignant changes in lung adenocarcinoma. Targeting VEGFA, TIMP1, and SPP1 may be a potential therapeutic strategy for lung adenocarcinoma.

Next, the same method of cell interaction network construction was applied to lung squamous cell carcinoma (LUSC), another type of non-small cell lung cancer. We observed that although the cell composition was similar to that of LUAD, the proportion of cells varied greatly from that of LUAD during the process of change from healthy to early to advanced stage, and the proportion of macrophages tended to increase. Further, by constructing a gene regulatory network for LUSC, we found that there is a common ligand signal GAS6 in tumor progression, but the target genes regulated by GAS6 are obviously different.

Altogether, the results of the validation group further verified the accuracy of our gene regulatory network construction method. The construction of a gene regulatory network is feasible for discovering important signals in cell-cell communication, and also provides a reference for screening the prognostic and therapeutic markers of patients.

## 4. Discussion

Lung adenocarcinoma is currently the leading cause of cancer death, and survival rates for lung cancer patients are poor. Lung adenocarcinoma tissues are highly heterogeneous. Single-cell RNA sequencing (scRNA-seq) analysis can provide a better understanding of cellular collective behavior and mutual regulatory mechanisms within a tissue ecosystem. In our study, transcriptional-level sequencing data of more than 135,000 single cells were collected, and various cell types were analyzed, providing a new perspective for understanding the cell composition characteristics and pathogenesis development in the LUAD.

The immune microenvironment of lung adenocarcinoma plays an important role in the initiation and development of lung adenocarcinoma. We found that the infiltration of macrophages in the immune microenvironment was significantly decreased with disease progression, and the function of macrophages was also significantly altered. In addition, we found, through survival analysis, low infiltration of macrophages is associated with poor survival in patients. Macrophages are an important part of the immune microenvironment. As the disease progresses, tumor-associated macrophage (TAM) subtypes play a dominant role in macrophage cells. TAMs exhibit an immunosuppressive protumor phenotype that promotes tumor progression, metastasis, and resistance to therapy [57,58]. The current study found that TAMs not only manipulate cancer cells toward progression and metastasis but also suppress the immune responses and cause chemoresistance [59]. Therefore, our results provided a supportive reference for the impact of macrophage cells on the occurrence and development of LUAD. It is worth noting that T cells (especially CD8 + T cells) play a very important role in the tumor microenvironment, but when we calculated the proportion of cells, we found that the proportion of T-cell infiltration did not change significantly in the lung microenvironment of patients during the process of change from healthy to early to advanced lung cancer. Moreover, by calculating T-cell immunoinfiltration with prognostic survival of lung adenocarcinoma patients, no significant relationship was found between the proportion of T-cell immunoinfiltration and the survival time of patients. We speculate that the total number of T cells may not have changed, but their function and composition have changed, based on the following experiment. T cells in normal tissues highly expressed T-effector cell markers, but tumor-infiltration of LUAD T cells mainly displayed exhausted and regulatory T-cell features, indicating compromised T-cytotoxic activity [60]. CD4 + T and regulatory T (Treg) cells associated with immune suppression and CD8 + exhausted cells were enriched in the LUAD, showing a shift in T-cell composition and gene expression towards immune suppression during LUAD progression [23].

Cell-cell communication influences cell phenotype and function [61]. At present, most of the cell-cell communication is ligand-receptor based. The construction of a gene regulatory network further elaborates the process of signal transmission between cell-cell communication and improves the integrity of cell-cell communication. In addition, the analytical methods of different cell-cell communication tools lead to different scores that are difficult to compare and evaluate. These methods make it difficult to validate the results [51,56]. We used several cell communication tools to evaluate consistency to improve the accuracy and confidence of signaling molecules. Based on the regulatory network analysis, we identified a number of ligands that have important regulatory effects on macrophage function, for instance, VEGFA, TIMP1, SPP1. It was demonstrated that TIMP1 could be a potential biomarker for LUAD pathogenesis through a protein fluorescence immunostaining experiment [24], promoting angiogenesis through the VEGFA signaling pathway and downstream target molecules in LUAD [62]; SPP1 plays a crucial role in mediating macrophage polarization and lung cancer escape, suggesting that these molecules are potential therapeutic targets for LUAD [63].

Similar results were obtained in independent verification sets for the regulatory relationships between the above regulatory molecules. First, we selected the same technical platform for (10× genomics) lung adenocarcinoma single-cell data for analysis using the same process, thereby reducing the error introduced by the different platforms. In the validation set, VEGFA, TIMP1 and SPP1 were still found to be significantly associated with the survival of lung adenocarcinoma patients. Both lung adenocarcinoma (LUAD) and lung squamous cell carcinoma (LUSC) are subtypes of non-small cell lung cancer (NSCLC), and we then applied the process to the LUSC single cell RNA-seq datasets from 10× genomics platform. We observed that although there were some similarities between the different NSCLC subtypes, there is also a great deal of heterogeneity due to their different tumor microenvironments. 

## 5. Conclusions

In conclusion, the construction of gene regulatory networks provides a comprehensive view of intercellular communication, which not only enhances the understanding of the molecular integrity of cell communication, but also provides a more credible signal transmission relationship. Important regulatory molecules (VEGFA, TIMP1, SPP1, etc.) of macrophages in lung adenocarcinoma were identified by the gene regulatory network method. Targeting VEGFA, TIMP1, and SPP1 may be potential therapeutic strategies for LUAD. Our research provides evidence of the tumor ecosystem heterogeneity between tLung and tL/B, in terms of cell proportion, macrophage developmental trajectories, in addition to the crosstalk between tumor and macrophages. Moreover, we have illustrated a strategy for constructing gene regulatory networks based on the cell-cell communication analysis of single-cell RNA sequencing data. We proved that such regulatory networks provide robust intercellular regulatory signals in the tumor microenvironment. This strategy may be applied to other cancer studies by single-cell RNA sequencing technology. The exploration of cell-cell communication by way of gene regulatory networks may lead to the discovery of novel therapeutic targets and biomarkers of response for current immunotherapies for LUAD and other cancers.

## Data Availability

The datasets presented in this study can be found in online repositories. The names of the repository/repositories and accession number(s) can be found in the article/Supplementary Material.

## Supplementary Materials

The following supporting information can be downloaded at: https://www.mdpi.com/article/10.3390/biom13040671/s1, Figure S1: (A) cell types of nLung, tLung and tL/B were identified by singleR. (B) Copy number variation of AT1 cell, ciliated cell, club cell, proliferating cell. The distant normal tissue (nLung) was used as reference, the early-stage LUAD (tLung), and the advanced stage LUAD (tL/B) were used as observation groups; red represents overexpression of genes and blue represents low expression. (C) The violin plot tumor markers of LUAD malignant cells. (D) The heatmap plot the correlation between different epithelial cells. Figure S2: (A) different percentages of cells in the sample (Patient_id_Source). (B) Box plot of the cell percentage of different immune cell types in three sources: distant normal tissue(nLung), early LUAD (tLung), and the advanced LUAD (tL/B). * p. adjust < 0.05, ** p. adjust < 0.01, and *** p. adjust < 0.001 (t-test). Figure S3: (A) correlation between the proportion of macrophages with survival rate, orange represents high proportion, green represents low proportion, (M0 macrophage, *p* = 0.039; M1 macrophage, *p* = 0.0013; M2 macrophage, *p* = 0.12). (B) The GO function enrichment analysis results of upregulated genes of the early LUAD (tLung) (distant normal tissue (nLung) as the control), and upregulated genes of the advanced LUAD (tL/B) (early LUAD (tLung) as the control), p. adjust < 0.05. Figure S4: (A) ligands differentially expressed between nLung and tLung, (B) downstream signals differentially expressed between nLung and tLung, (C) ligands differentially expressed between tLung and tL/B, (D) downstream signals differentially expressed between tLung and tL/B. Figure S5: (A–D) the GO function enrichment analysis results of states 3, 4, 5, and 6 macrophages. Figure S6: (A) the umap plot clusters of macrophages in nLung, tLung and tL/B. (B) The violin plot tumor-associated macrophage markers of different clusters. (C) The violin plot alveolar macrophage markers of different clusters. (D) The umap plot cell types of macrophages in nLung, tLung and tL/B. (E) The umap plot states of macrophages in nLung, tLung and tL/B. Figure S7: (A) a single-cell atlas of nLung, tLung and tL/B in LUSC. (B) Proportion of cells in nLung, tLung and tL/B. (C) Changes in regulatory networks in nLung and tLung. (D) Changes in regulatory networks in tLung and tL/B. Table S1: Clinical information from single-cell transcriptome data of lung adenocarcinoma patients.

## References

[1] Seguin L., Durandy M., Feral C.C. (2022). Lung Adenocarcinoma Tumor Origin: A Guide for Personalized Medicine. Cancers.

[2] Torre L.A., Siegel R.L., Jemal A. (2016). Lung Cancer Statistics. Adv. Exp. Med. Biol..

[3] Wang B.Y., Huang J.Y., Chen H.C., Lin C.H., Lin S.H., Hung W.H., Cheng Y.F. (2020). The comparison between adenocarcinoma and squamous cell carcinoma in lung cancer patients. J. Cancer Res. Clin. Oncol..

[4] Zhang L., Zhang Y., Wang C., Yang Y., Ni Y., Wang Z., Song T., Yao M., Liu Z., Chao N. (2022). Integrated single-cell RNA sequencing analysis reveals distinct cellular and transcriptional modules associated with survival in lung cancer. Signal Transduct. Target. Ther..

[5] Wang C., Yu Q., Song T., Wang Z., Song L., Yang Y., Shao J., Li J., Ni Y., Chao N. (2022). The heterogeneous immune landscape between lung adenocarcinoma and squamous carcinoma revealed by single-cell RNA sequencing. Signal Transduct. Target. Ther..

[6] Khullar O.V., Liu Y., Gillespie T., Higgins K.A., Ramalingam S., Lipscomb J., Fernandez F.G. (2015). Survival After Sublobar Resection versus Lobectomy for Clinical Stage IA Lung Cancer: An Analysis from the National Cancer Data Base. J. Thorac. Oncol. Off. Publ. Int. Assoc. Study Lung Cancer.

[7] Hanna N., Johnson D., Temin S., Baker S., Brahmer J., Ellis P.M., Giaccone G., Hesketh P.J., Jaiyesimi I., Leighl N.B. (2017). Systemic Therapy for Stage IV Non-Small-Cell Lung Cancer: American Society of Clinical Oncology Clinical Practice Guideline Update. J. Clin. Oncol. Off. J. Am. Soc. Clin. Oncol..

[8] Friemel J., Frick L., Weber A. (2015). Intratumor heterogeneity in HCC. Aging.

[9] Navin N., Krasnitz A., Rodgers L., Cook K., Meth J., Kendall J., Riggs M., Eberling Y., Troge J., Grubor V. (2010). Inferring tumor progression from genomic heterogeneity. Genome Res..

[10] Cohen M., Giladi A., Gorki A.D., Solodkin D.G., Zada M., Hladik A., Miklosi A., Salame T.M., Halpern K.B., David E. (2018). Lung Single-Cell Signaling Interaction Map Reveals Basophil Role in Macrophage Imprinting. Cell.

[11] Boisset J.C., Vivié J., Grün D., Muraro M.J., Lyubimova A., van Oudenaarden A. (2018). Mapping the physical network of cellular interactions. Nat. Methods.

[12] Qiao W., Wang W., Laurenti E., Turinsky A.L., Wodak S.J., Bader G.D., Dick J.E., Zandstra P.W. (2014). Intercellular network structure and regulatory motifs in the human hematopoietic system. Mol. Syst. Biol..

[13] Paik D.T., Tian L., Lee J., Sayed N., Chen I.Y., Rhee S., Rhee J.W., Kim Y., Wirka R.C., Buikema J.W. (2018). Large-Scale Single-Cell RNA-Seq Reveals Molecular Signatures of Heterogeneous Populations of Human Induced Pluripotent Stem Cell-Derived Endothelial Cells. Circ. Res..

[14] Kumar M.P., Du J., Lagoudas G., Jiao Y., Sawyer A., Drummond D.C., Lauffenburger D.A., Raue A. (2018). Analysis of Single-Cell RNA-Seq Identifies Cell-Cell Communication Associated with Tumor Characteristics. Cell Rep..

[15] Tirosh I., Izar B., Prakadan S.M., Wadsworth M.H., Treacy D., Trombetta J.J., Rotem A., Rodman C., Lian C., Murphy G. (2016). Dissecting the multicellular ecosystem of metastatic melanoma by single-cell RNA-seq. Science.

[16] Martin J.C., Chang C., Boschetti G., Ungaro R., Giri M., Grout J.A., Gettler K., Chuang L.S., Nayar S., Greenstein A.J. (2019). Single-Cell Analysis of Crohn’s Disease Lesions Identifies a Pathogenic Cellular Module Associated with Resistance to Anti-TNF Therapy. Cell.

[17] AlMusawi S., Ahmed M., Nateri A.S. (2021). Understanding cell-cell communication and signaling in the colorectal cancer microenvironment. Clin. Transl. Med..

[18] Hinshaw D.C., Shevde L.A. (2019). The Tumor Microenvironment Innately Modulates Cancer Progression. Cancer Res..

[19] Chang R.B., Beatty G.L. (2020). The interplay between innate and adaptive immunity in cancer shapes the productivity of cancer immunosurveillance. J. Leukoc. Biol..

[20] Wei K., Ma Z., Yang F., Zhao X., Jiang W., Pan C., Li Z., Pan X., He Z., Xu J. (2022). M2 macrophage-derived exosomes promote lung adenocarcinoma progression by delivering miR-942. Cancer Lett..

[21] Wu J., Zhou J., Xu Q., Foley R., Guo J., Zhang X., Tian C., Mu M., Xing Y., Liu Y. (2021). Identification of Key Genes Driving Tumor Associated Macrophage Migration and Polarization Based on Immune Fingerprints of Lung Adenocarcinoma. Front. Cell Dev. Biol..

[22] Bischoff P., Trinks A., Obermayer B., Pett J.P., Wiederspahn J., Uhlitz F., Liang X., Lehmann A., Jurmeister P., Elsner A. (2021). Single-cell RNA sequencing reveals distinct tumor microenvironmental patterns in lung adenocarcinoma. Oncogene.

[23] Wang Z., Li Z., Zhou K., Wang C., Jiang L., Zhang L., Yang Y., Luo W., Qiao W., Wang G. (2021). Deciphering cell lineage specification of human lung adenocarcinoma with single-cell RNA sequencing. Nat. Commun..

[24] Sinjab A., Han G., Treekitkarnmongkol W., Hara K., Brennan P.M., Dang M., Hao D., Wang R., Dai E., Dejima H. (2021). Resolving the Spatial and Cellular Architecture of Lung Adenocarcinoma by Multiregion Single-Cell Sequencing. Cancer Discov..

[25] Chen Z., Huang Y., Hu Z., Zhao M., Li M., Bi G., Zheng Y., Liang J., Lu T., Jiang W. (2021). Landscape and dynamics of single tumor and immune cells in early and advanced-stage lung adenocarcinoma. Clin. Transl. Med..

[26] Kim N., Kim H.K., Lee K., Hong Y., Cho J.H., Choi J.W., Lee J.I., Suh Y.L., Ku B.M., Eum H.H. (2020). Single-cell RNA sequencing demonstrates the molecular and cellular reprogramming of metastatic lung adenocarcinoma. Nat. Commun..

[27] Laughney A.M., Hu J., Campbell N.R., Bakhoum S.F., Setty M., Lavallée V.P., Xie Y., Masilionis I., Carr A.J., Kottapalli S. (2020). Regenerative lineages and immune-mediated pruning in lung cancer metastasis. Nat. Med..

[28] Wang F., Zhang Y., Hao Y., Li X., Qi Y., Xin M., Xiao Q., Wang P. (2021). Characterizing the Metabolic and Immune Landscape of Non-small Cell Lung Cancer Reveals Prognostic Biomarkers Through Omics Data Integration. Front. Cell Dev. Biol..

[29] Wu F., Fan J., He Y., Xiong A., Yu J., Li Y., Zhang Y., Zhao W., Zhou F., Li W. (2021). Single-cell profiling of tumor heterogeneity and the microenvironment in advanced non-small cell lung cancer. Nat. Commun..

[30] Korsunsky I., Millard N., Fan J., Slowikowski K., Zhang F., Wei K., Baglaenko Y., Brenner M., Loh P.R., Raychaudhuri S. (2019). Fast, sensitive and accurate integration of single-cell data with Harmony. Nat. Methods.

[31] Butler A., Hoffman P., Smibert P., Papalexi E., Satija R. (2018). Integrating single-cell transcriptomic data across different conditions, technologies, and species. Nat. Biotechnol..

[32] Aran D., Looney A.P., Liu L., Wu E., Fong V., Hsu A., Chak S., Naikawadi R.P., Wolters P.J., Abate A.R. (2019). Reference-based analysis of lung single-cell sequencing reveals a transitional profibrotic macrophage. Nat. Immunol..

[33] Zhang X., Lan Y., Xu J., Quan F., Zhao E., Deng C., Luo T., Xu L., Liao G., Yan M. (2019). CellMarker: A manually curated resource of cell markers in human and mouse. Nucleic Acids Res..

[34] Kumar M., Bowers R.R., Delaney J.R. (2020). Single-cell analysis of copy-number alterations in serous ovarian cancer reveals substantial heterogeneity in both low- and high-grade tumors. Cell Cycle (Georget. Tex.).

[35] Yu G., Wang L.G., Han Y., He Q.Y. (2012). clusterProfiler: An R package for comparing biological themes among gene clusters. Omics A J. Integr. Biol..

[36] Kanehisa M., Furumichi M., Tanabe M., Sato Y., Morishima K. (2017). KEGG: New perspectives on genomes, pathways, diseases and drugs. Nucleic Acids Res..

[37] Chen B., Khodadoust M.S., Liu C.L., Newman A.M., Alizadeh A.A. (2018). Profiling Tumor Infiltrating Immune Cells with CIBERSORT. Methods Mol. Biol..

[38] George B., Seals S., Aban I. (2014). Survival analysis and regression models. J. Nucl. Cardiol. Off. Publ. Am. Soc. Nucl. Cardiol..

[39] Tang Z., Kang B., Li C., Chen T., Zhang Z. (2019). GEPIA2: An enhanced web server for large-scale expression profiling and interactive analysis. Nucleic Acids Res..

[40] Browaeys R., Saelens W., Saeys Y. (2020). NicheNet: Modeling intercellular communication by linking ligands to target genes. Nat. Methods.

[41] Efremova M., Vento-Tormo M., Teichmann S.A., Vento-Tormo R. (2020). CellPhoneDB: Inferring cell-cell communication from combined expression of multi-subunit ligand-receptor complexes. Nat. Protoc..

[42] Kumar N., Mishra B., Athar M., Mukhtar S. (2021). Inference of Gene Regulatory Network from Single-Cell Transcriptomic Data Using pySCENIC. Methods Mol. Biol..

[43] Trapnell C., Cacchiarelli D., Grimsby J., Pokharel P., Li S., Morse M., Lennon N.J., Livak K.J., Mikkelsen T.S., Rinn J.L. (2014). The dynamics and regulators of cell fate decisions are revealed by pseudotemporal ordering of single cells. Nat. Biotechnol..

[44] Lin Y., Chen D., Ding Q., Zhu X., Zhu R., Chen Y. (2021). Progress in Single-cell RNA Sequencing of Lung Adenocarcinoma. Zhongguo Fei Ai Za Zhi = Chin. J. Lung Cancer.

[45] Kurtenbach S., Cruz A.M., Rodriguez D.A., Durante M.A., Harbour J.W. (2021). Uphyloplot2: Visualizing phylogenetic trees from single-cell RNA-seq data. BMC Genom..

[46] Liu Y., Ye G., Huang L., Zhang C., Sheng Y., Wu B., Han L., Wu C., Dong B., Qi Y. (2020). Single-cell transcriptome analysis demonstrates inter-patient and intra-tumor heterogeneity in primary and metastatic lung adenocarcinoma. Aging.

[47] Sheu K.M., Hoffmann A. (2022). Functional Hallmarks of Healthy Macrophage Responses: Their Regulatory Basis and Disease Relevance. Annu. Rev. Immunol..

[48] Zhao R., Cao J., Yang X., Zhang Q., Iqbal M.Z., Lu J., Kong X. (2021). Inorganic material based macrophage regulation for cancer therapy: Basic concepts and recent advances. Biomater. Sci..

[49] Zhou L., Xu G., Wang L., Zhang J., Li W. (2021). Derivation of a Novel CIHI in Patients with Lung Adenocarcinoma for Estimating Tumor Microenvironment and Clinical Prognosis. Dis. Mrk..

[50] Dong Y., Arif A.A., Guo J., Ha Z., Lee-Sayer S.S.M., Poon G.F.T., Dosanjh M., Roskelley C.D., Huan T., Johnson P. (2020). CD44 Loss Disrupts Lung Lipid Surfactant Homeostasis and Exacerbates Oxidized Lipid-Induced Lung Inflammation. Front. Immunol..

[51] Viswanadhapalli S., Dileep K.V., Zhang K.Y.J., Nair H.B., Vadlamudi R.K. (2022). Targeting LIF/LIFR signaling in cancer. Genes Dis..

[52] Liao J., Hargreaves D.C. (2020). The alternative macrophage relay: STAT6 passes the baton to EGR2. Genes Dev..

[53] Zhang S., Liu Z., Wu D., Chen L., Xie L. (2020). Single-Cell RNA-Seq Analysis Reveals Microenvironmental Infiltration of Plasma Cells and Hepatocytic Prognostic Markers in HCC With Cirrhosis. Front. Oncol..

[54] Korbecki J., Simińska D., Gąssowska-Dobrowolska M., Listos J., Gutowska I., Chlubek D., Baranowska-Bosiacka I. (2021). Chronic and Cycling Hypoxia: Drivers of Cancer Chronic Inflammation through HIF-1 and NF-κB Activation: A Review of the Molecular Mechanisms. Int. J. Mol. Sci..

[55] Justo B.L., Jasiulionis M.G. (2021). Characteristics of TIMP1, CD63, and β1-Integrin and the Functional Impact of Their Interaction in Cancer. Int. J. Mol. Sci..

[56] Tang H., Chen J., Han X., Feng Y., Wang F. (2021). Upregulation of SPP1 Is a Marker for Poor Lung Cancer Prognosis and Contributes to Cancer Progression and Cisplatin Resistance. Front. Cell Dev. Biol..

[57] Lopez-Yrigoyen M., Cassetta L., Pollard J.W. (2021). Macrophage targeting in cancer. Ann. N. Y. Acad. Sci..

[58] Wu K., Lin K., Li X., Yuan X., Xu P., Ni P., Xu D. (2020). Redefining Tumor-Associated Macrophage Subpopulations and Functions in the Tumor Microenvironment. Front. Immunol..

[59] Cha Y.J., Koo J.S. (2020). Role of Tumor-Associated Myeloid Cells in Breast Cancer. Cells.

[60] He D., Wang D., Lu P., Yang N., Xue Z., Zhu X., Zhang P., Fan G. (2021). Single-cell RNA sequencing reveals heterogeneous tumor and immune cell populations in early-stage lung adenocarcinomas harboring EGFR mutations. Oncogene.

[61] Almet A.A., Cang Z., Jin S., Nie Q. (2021). The landscape of cell-cell communication through single-cell transcriptomics. Curr. Opin. Syst. Biol..

[62] Wang Y., Zhang F., Wang J., Hu L., Jiang F., Chen J., Chen J., Wang L. (2018). lncRNA LOC100132354 promotes angiogenesis through VEGFA/VEGFR2 signaling pathway in lung adenocarcinoma. Cancer Manag. Res..

[63] Zhang Y., Du W., Chen Z., Xiang C. (2017). Upregulation of PD-L1 by SPP1 mediates macrophage polarization and facilitates immune escape in lung adenocarcinoma. Exp. Cell Res..

