# Supplementary material for: Single-Cell RNA Sequencing Analysis of Gene Regulatory Network Changes in the Development of Lung Adenocarcinoma
Source: Biomolecules. 2023 Apr 12;13(4):671. doi: 10.3390/biom13040671 (PMC10135828; doi:10.3390/biom13040671)

Supplementary Figure S1

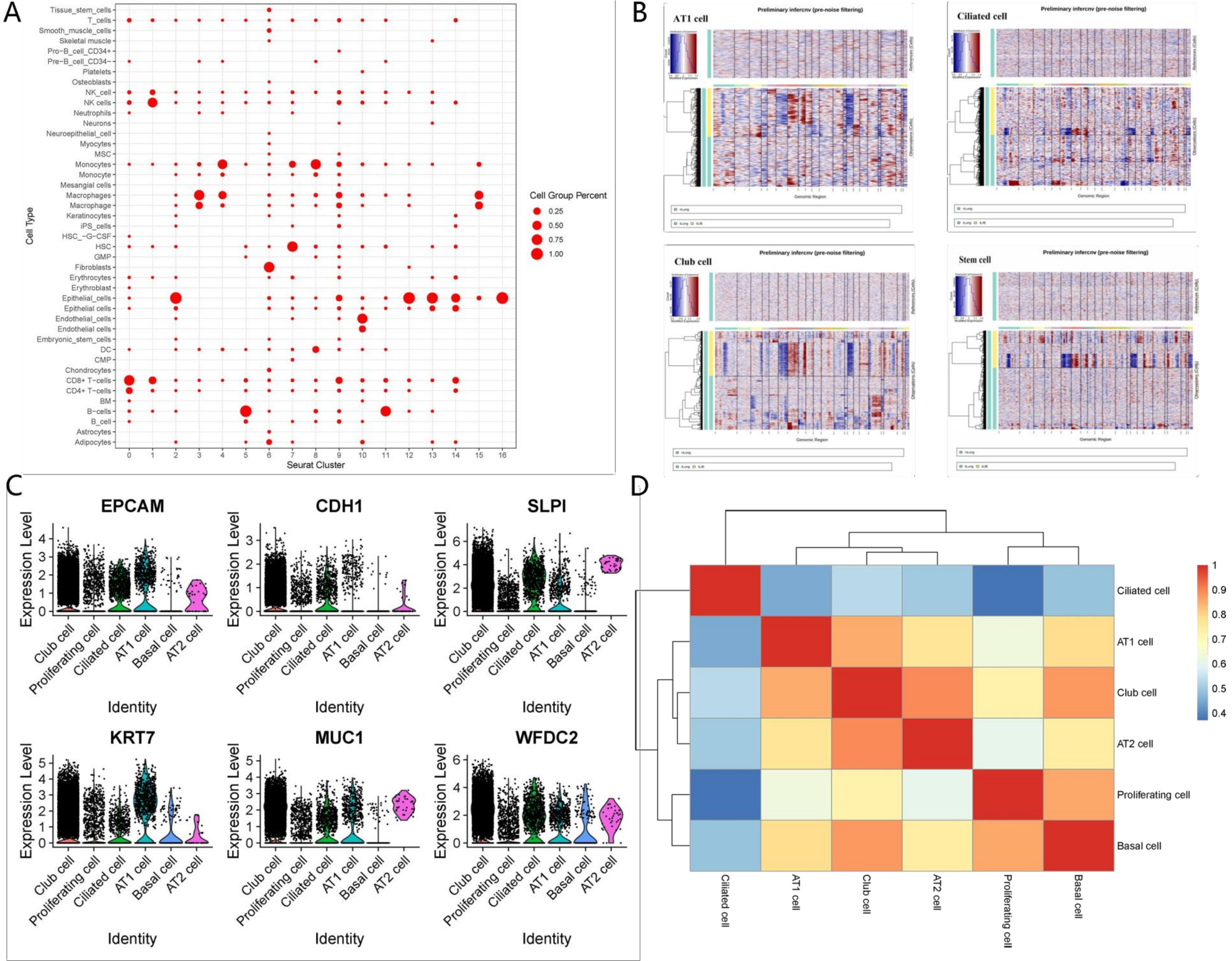

Supplementary Figure S2

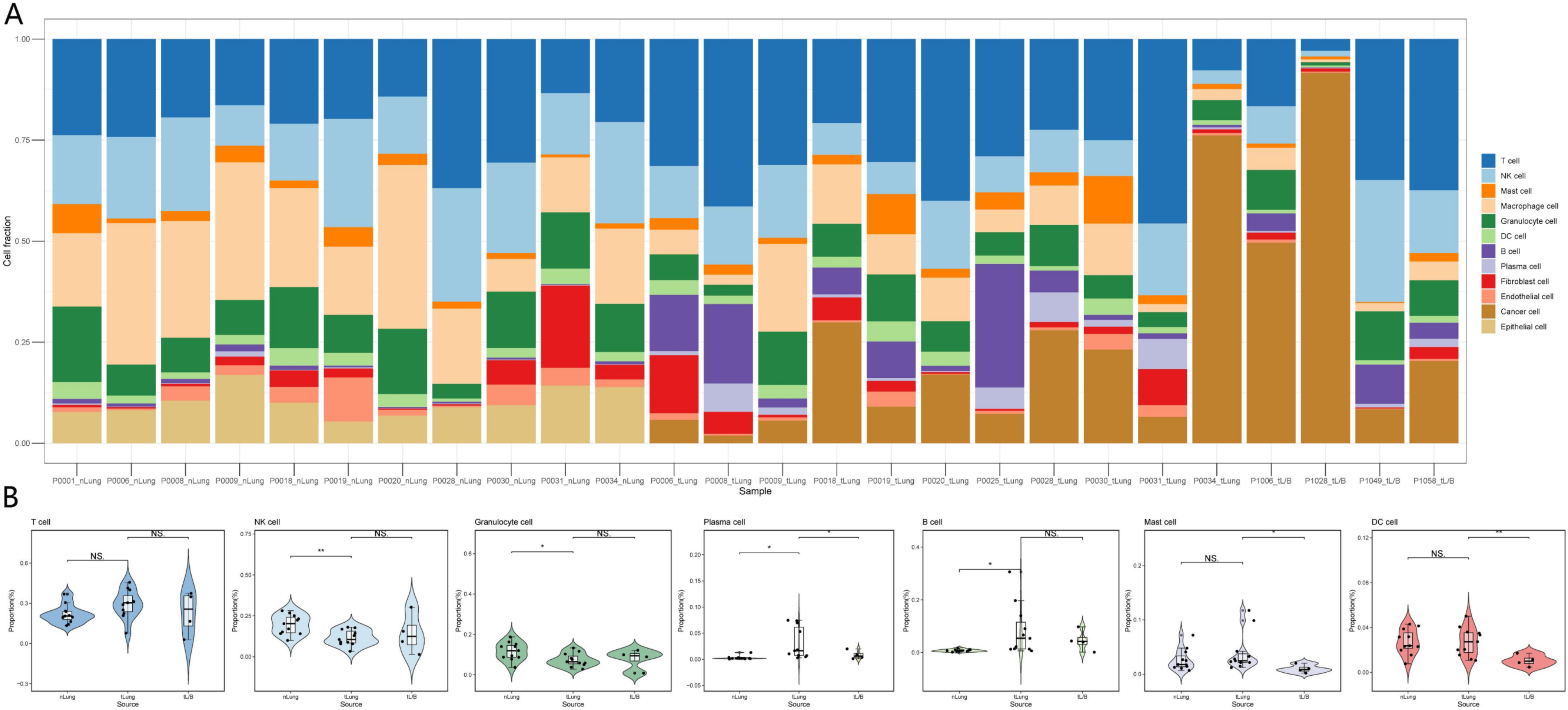

Supplementary Figure S3

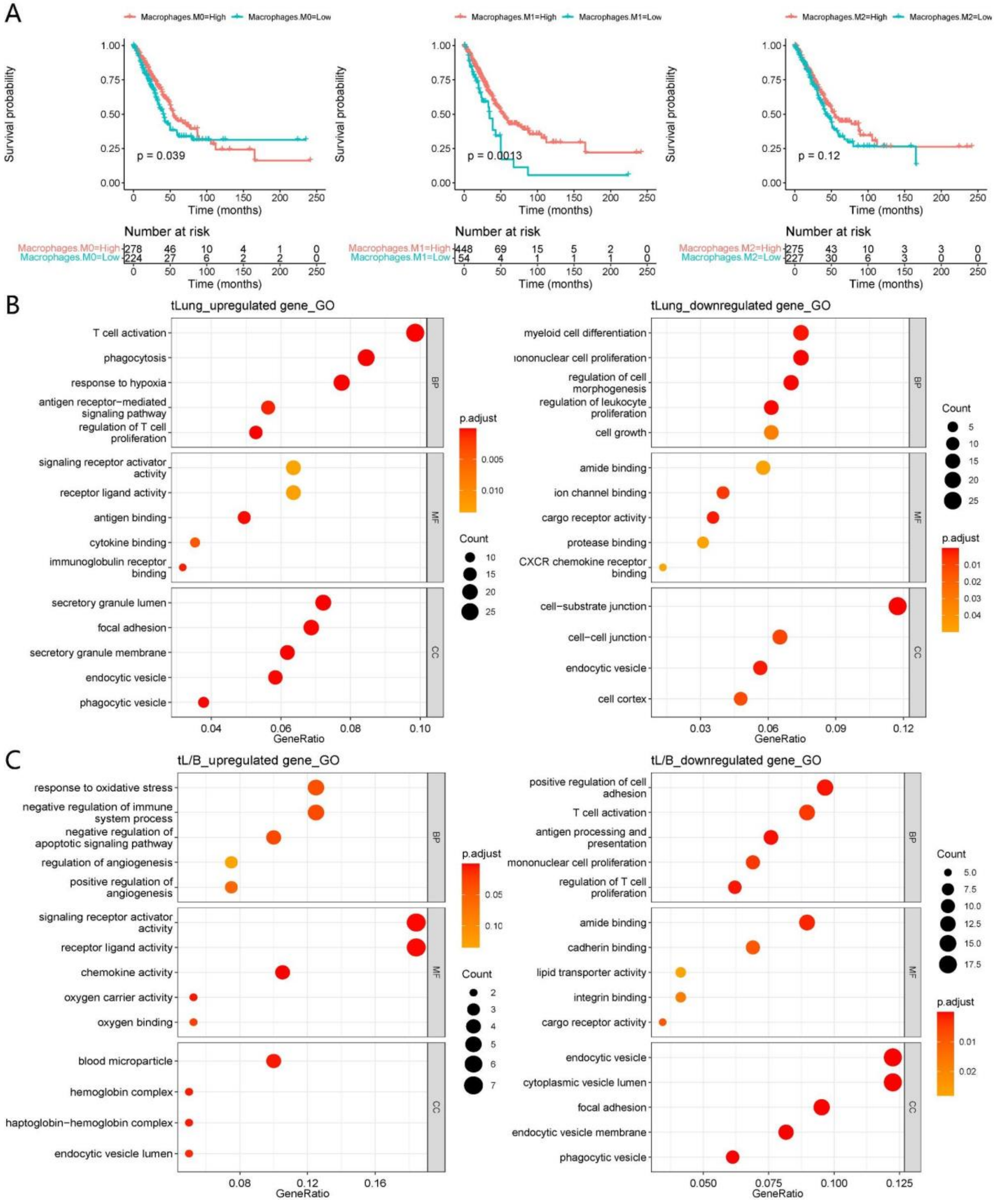

Supplementary Figure S4

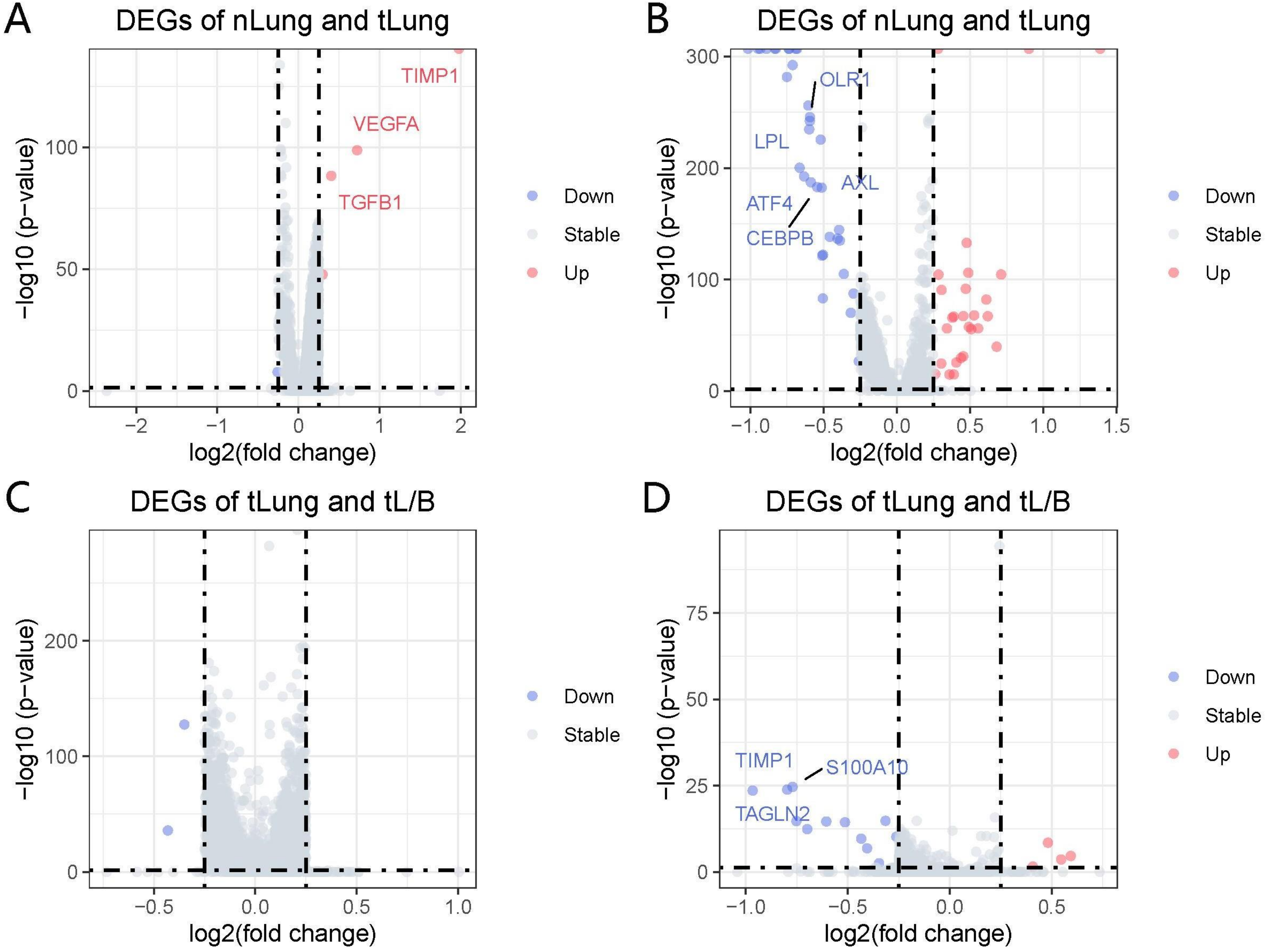

Supplementary Figure S5

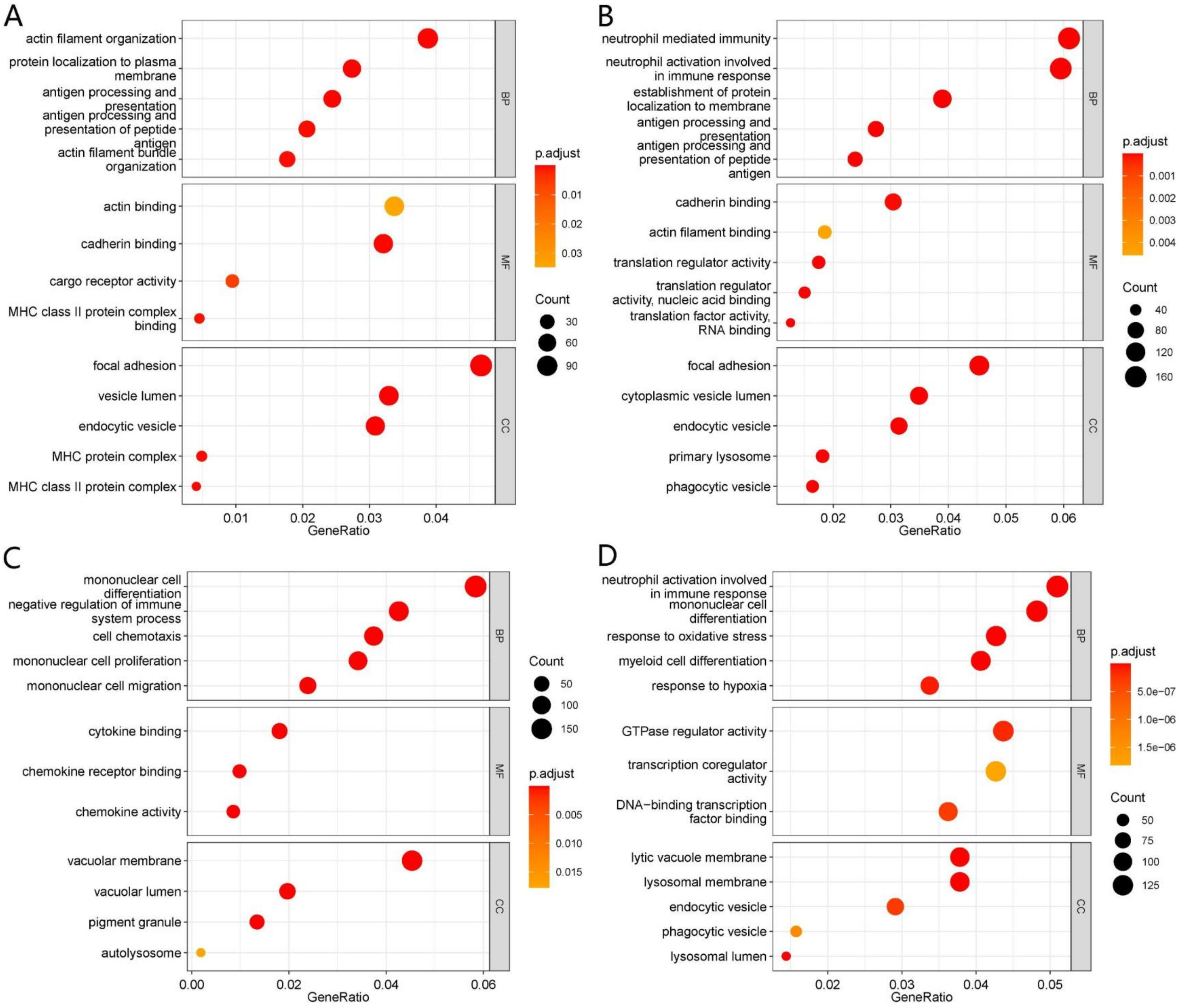

Supplementary Figure S6

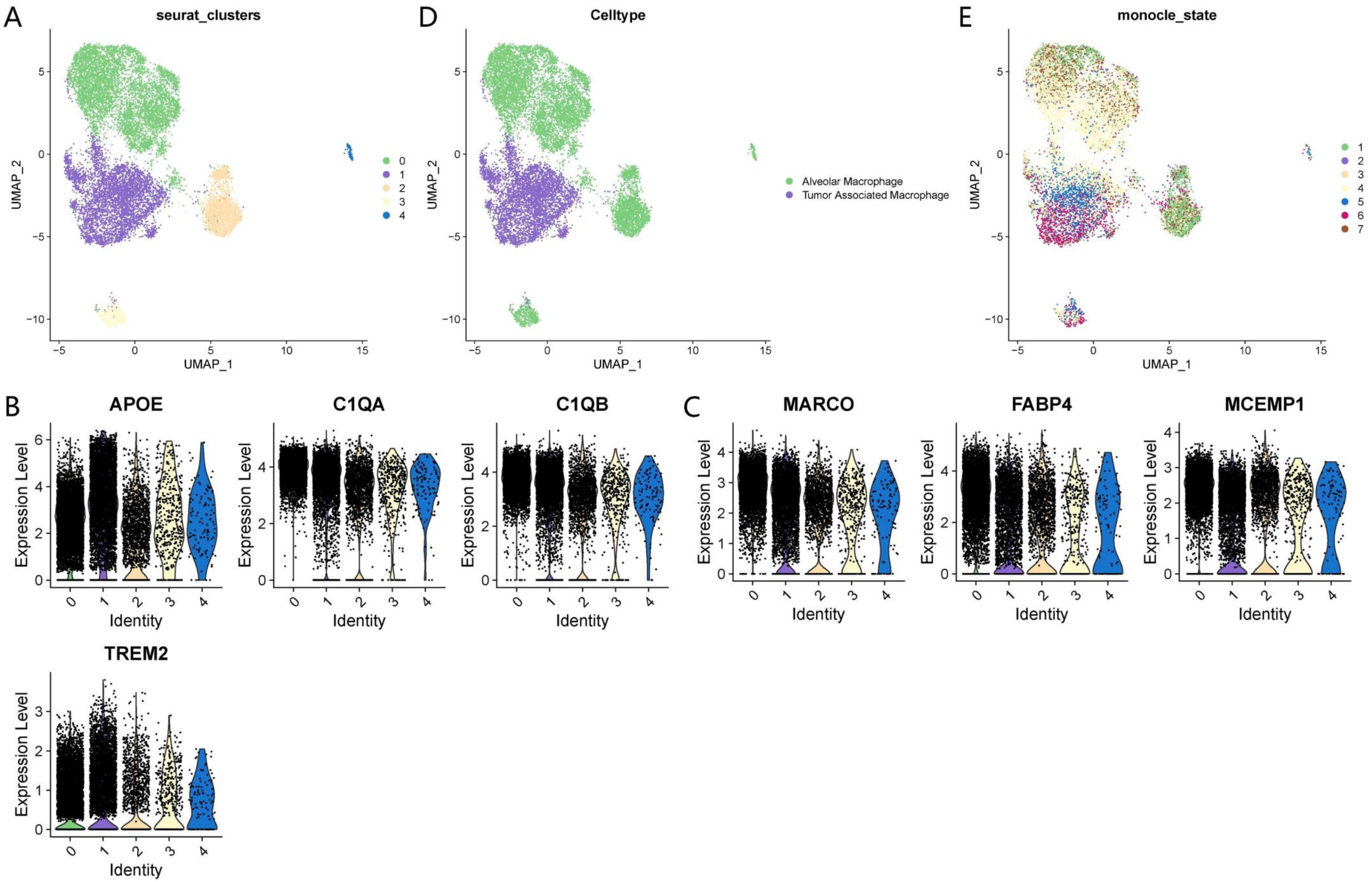

Supplementary Figure S7

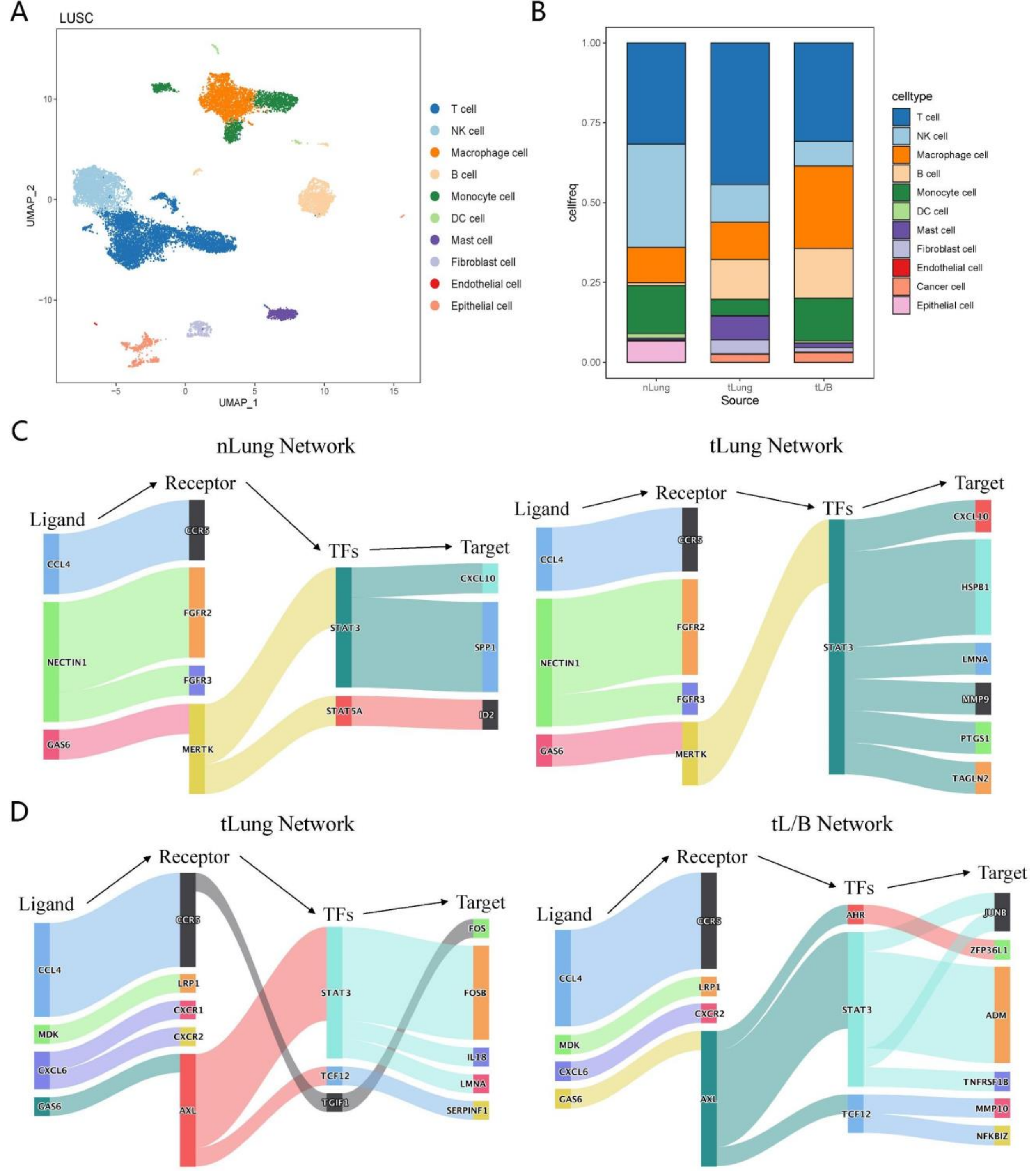

Supplement: Supplementary file 1 [file biomolecules-13-00671-s001.zip › Supplementary_Figures.pdf]
