# Supplementary material for: Single-Cell RNA Sequencing Analysis of Gene Regulatory Network Changes in the Development of Lung Adenocarcinoma
Source: Biomolecules. 2023 Apr 12;13(4):671. doi: 10.3390/biom13040671 (PMC10135828; doi:10.3390/biom13040671)
Supplement: Supplementary file 1 [file biomolecules-13-00671-s001.zip › Supplementary_Table S1.pdf]

Supplementary Table S1

Feature summary of the lung adenocarcinoma specimens and single-cell RNA sequencing data

No	Patient id	Samples	Tissue origins	Histology	Smoking	Pathology	EGFR	Stages
1	P0001	LUNG_N01	nLung	ADC	Never	MD	WT	IA
2	P0006	LUNG_N06	nLung	ADC	Ex	MD	na	IA
3	P0008	LUNG_N08	nLung	ADC	Never	MD	L858R	IB
4	P0009	LUNG_N09	nLung	ADC	Ex	PD	WT	IIA
5	P0018	LUNG_N18	nLung	ADC	Ex	MD	del19	IA
6	P0019	LUNG_N19	nLung	ADC	Cur	WD	exon 20	IA
7	P0020	LUNG_N20	nLung	ADC	Cur	PD	WT	IA
8	P0028	LUNG_N28	nLung	ADC(Double)	Cur	na	WT	IIIA
9	P0030	LUNG_N30	nLung	ADC	Never	na	del19	IA
10	P0031	LUNG_N31	nLung	ADC	Ex	na	WT	IIIA
11	P0034	LUNG_N34	nLung	ADC	Never	MD	WT	IA3
12	P0006	LUNG_T06	tLung	ADC	Ex	MD	na	IA
13	P0008	LUNG_T08	tLung	ADC	Never	MD	L858R	IB
14	P0009	LUNG_T09	tLung	ADC	Ex	PD	WT	IIA
15	P0018	LUNG_T18	tLung	ADC	Ex	MD	del19	IA
16	P0019	LUNG_T19	tLung	ADC	Cur	WD	exon 20	IA
17	P0020	LUNG_T20	tLung	ADC	Cur	PD	WT	IA
18	P0025	LUNG_T25	tLung	ADC(Double)	Ex	na	WT	IA
19	P0028	LUNG_T28	tLung	ADC(Double)	Cur	na	WT	IIIA
20	P0030	LUNG_T30	tLung	ADC	Never	na	del19	IA
21	P0031	LUNG_T31	tLung	ADC	Ex	na	WT	IIIA
22	P0034	LUNG_T34	tLung	ADC	Never	MD	WT	IA3
23	P1006	EBUS_06	tL/B	ADC	Cur	PD	WT	IV
24	P1028	EBUS_28	tL/B	ADC	Ex	na	WT	IV
25	P1049	EBUS_49	tL/B	ADC	Cur	PD	WT	IV
26	P1058	BRONCHO_58	tL/B	ADC	Never	PD	na	IV

nLung, normal lung; tLung, early stage tumor lung; tL/B, advanced stage tumor lung;ADC, Adenocarcinoma; Cur, Current smoker; Ex, Ex-smoker; Never, Never smoker; PD, poorly differentiated; MD, moderately differentiated; WD, well differentiated;
